# Long-Acting Metformin Vs. Metformin Immediate Release in Patients With Type 2 Diabetes: A Systematic Review

**DOI:** 10.3389/fphar.2021.669814

**Published:** 2021-05-17

**Authors:** Jixue Tan, Yang Wang, Song Liu, Qingyang Shi, Xu Zhou, Yiling Zhou, Xiaoling Yang, Pingshan Chen, Sheyu Li

**Affiliations:** ^1^ The Second School of Clinical Medicine, Nanchang University, Nanchang, China; ^2^ Department of Science and Technology, The Second Affiliated Hospital of Nanchang University, Nanchang, China; ^3^ Queen Mary School, Nanchang University, Nanchang, China; ^4^ Department of Endocrinology and Metabolism, West China Hospital, Sichuan University, Chengdu, China; ^5^ West China School of Medicine, Sichuan University, Chengdu, China; ^6^ Department of Guideline and Rapid Recommendation, Cochrane China Center, MAGIC China Center, Chinese Evidence-Based Medicine Center, West China Hospital, Sichuan University, Chengdu, China; ^7^ Evidence-Based Medicine Research Center, Jiangxi University of Traditional Chinese Medicine, Nanchang, China

**Keywords:** metformin immediate-release, metformin extended-release, treatment compliance, once-daily consumption, systematic review, meta-analysis, patient value

## Abstract

**Background:** Metformin, a commonly used antidiabetic medication, is available in both an immediate-release (IR) formulation and a long-acting formulation (metformin extended-release; XR).

**Objective:** We performed a systematic review to compare the effectiveness, safety, and patient compliance and satisfaction between the metformin IR and XR formulations.

**Method:** We searched for randomized control trials (RCTs) and observational studies comparing the effectiveness, safety, or patient compliance and satisfaction of metformin XR with metformin IR using the MEDLINE, Embase, and Cochrane Central Register of Controlled Trials databases. Following report screening, data collection, and risk of bias assessment, we separately pooled data from RCTs and observational studies using the Grading of Recommendation Assessment, Development, and Evaluation approach to rate the quality of evidence.

**Result:** We included five RCTs, comprising a total of 1,662 patients, and one observational study, comprising 10,909 patients. In the meta-analyses, no differences were identified in outcomes of effectiveness and safety between the two forms of metformin (including change in HbA1c: mean difference (MD), 0.04%, 95% confidence interval [CI], −0.05–0.13%, fasting blood glucose: MD, −0.03 mmol/L, 95% CI, −0.22–0.15 mmol/L, postprandial blood glucose: MD, 0.50 mmol/L, 95% CI, −0.71–1.72 mmol/L, adverse events of abdominal pain: relative risk (RR), 1.15, 95% CI, 0.57–2.33, all-cause death (RR, 3.02, 95% CI 0.12–73.85), any adverse events (RR, 1.14, 95% CI 0.97–1.34), any adverse events leading to treatment discontinuation: RR, 1.51, 95% CI, 0.82–2.8, any gastrointestinal adverse events: RR, 1.09, 95% CI, 0.93–1.29, diarrhea: RR, 0.82, 95% CI, 0.53–1.27, flatulence: RR, 0.43, 95% CI, 0.15–1.23, nausea: RR, 0.97, 95% CI, 0.64–1.47, severe adverse events: RR, 0.64, 95% CI, 0.28–1.42, and vomiting: RR, 1.46, 95% CI, 0.6–3.56). Data from both the RCTs and the observational study indicate mildly superior patient compliance with metformin XR use compared with metformin IR use; this result was attributable to the preference for once-daily administration with metformin XR.

**Conclusion:** Our systematic review indicates that metformin XR and IR formulations have similar effectiveness and safety, but that metformin XR is associated with improved compliance to treatment.

## Introduction

Type 2 diabetes mellitus was the fourth leading cause of death and disability worldwide in 2017, and has rapidly risen in rank since 1990 (Roth et al., 2018). Approximately 1 in 11 adults worldwide had type 2 diabetes, and those affected can experience life-threatening complications, including cardiovascular and renal diseases (Zheng et al., 2018). Lifestyle intervention and careful pharmacotherapeutic management are critical measures for people living with type 2 diabetes (Amod, 2012; McGuire et al., 2016).

Metformin effectively lowers blood glucose levels and is widely used to treat type 2 diabetes (National Collaborating Centre for Chronic Conditions (UK), 2008; Amod, 2012). Furthermore, metformin use is associated with long-term benefits, including reduced risk of cardiovascular disease and neoplasms (Foretz et al., 2014). Gastrointestinal intolerance is a major concern associated with clinical use of metformin; approximately 10% of adults living with type 2 diabetes are unable to receive metformin treatment because of gastrointestinal intolerance including diarrhea, vomiting, abdominal pain, and constipation (Fujioka et al., 2003; Ji et al., 2018). Long-term treatment with metformin is generally considered safe, except for its association with an increased risk of vitamin B12 deficiency (Reinstatler et al., 2012; Liu et al., 2014). However, the clinical relevance of such a deficiency remains unclear (Zhang et al., 2016).

Metformin tablets are available in an immediate-release (IR) formulation for administration two or three times per day. The need to administer the drug multiple times daily can present challenges for treatment compliance among patients and the maintenance of steady-state pharmacokinetics (Fujioka et al., 2003; Ji et al., 2018). An extended-release (XR) formulation with longer half-life and lower peak drug concentration is also available (Amod, 2012). Adults with type 2 diabetes receiving once-daily metformin XR might have a better treatment experience and are more likely to comply with treatment (Fujioka et al., 2003; Blonde et al., 2004).

The United Kingdom National Institute of Health and Care Excellence (NICE) guidelines recommend the use of metformin XR in adults with type 2 diabetes who cannot tolerate metformin IR (National Collaborating Centre for Chronic Conditions (UK), 2008); however, few studies have examined this recommendation. We previously reported the results of a survey conducted in adults living with type 2 diabetes and their physicians; we found that patients and physicians considered metformin XR to be superior to the IR formulation in terms of both effectiveness and safety (Liu et al., 2021). However, other studies have provided conflicting evidence (Fujioka et al., 2003, Blonde et al., 2004, National Collaborating Centre for Chronic Conditions (UK), 2008, Reinstatler et al., 2012, Foretz et al., 2014, Liu et al., 2014 ,Zhang et al., 2016, Ji et al., 2018). Data from a randomized controlled trial (RCT) of 267 patients in China indicated that the effectiveness and safety of metformin XR and IR were comparable (Ji et al., 2018), and these results have not been confirmed in other RCTs or observational studies (Blonde et al., 2004).

We therefore performed a systematic review comprising a comprehensive search and qualitative analyses to compare the short-term effectiveness and safety of metformin XR and IR in adults with type 2 diabetes.

## Methods

This systematic review was conducted in accordance with the Preferred Reporting Items for Systematic Reviews and Meta-Analyses (PRISMA) guidelines (Moher et al., 2011). The protocol of this systematic review was registered in PROSPERO (number: CRD42021226051).

### Literature Search

We systematically searched the MEDLINE, EMBASE, and Cochrane Central Register of Controlled Trials (CENTRAL) *via* OVID from study inception until September 26, 2020 using subject term and free keywords such as metformin XR and long-acting metformin. The supplementary material shows the details of the searching strategy. These searches were supplemented by a search of Clinicaltrials.gov for trials that were complete but not yet published. For duplicate results and studies with overlapping populations, only one report was included. When choosing which to include, we selected the one with the longest follow-up duration or the one with the largest population in cases where the follow-up duration was equal. The search strategy is shown in the supplementary information.

### Eligibility Criteria

Searches were limited to RCTs and observational studies comparing long-acting metformin (including metformin XR, controlled release, and sustained release) with metformin IR in adults with type 2 diabetes. We included studies published in English that had a follow-up duration of at least 3 months (or 12 weeks) and reported at least one of the outcomes of interest.

The following studies were excluded: cross-sectional surveys, case series, and case reports; studies in which patients were treated with systematically different doses in the XR and IR groups; and studies in pregnant women.

### Outcomes

Outcomes of interest were change from baseline in glycated hemoglobin (HbA1c), fasting plasma glucose (FPG), and postprandial blood glucose. Safety data including all-cause death, number of overall adverse events, adverse events leading to discontinuation, severe adverse events, and gastrointestinal adverse events were included, as were type of gastrointestinal event including vomiting, nausea, abdominal pain, flatulence, and diarrhea. Data on compliance to treatment and patient satisfaction were also included.

### Study Selection

Two reviewers (JT and YW) screened the titles and abstracts of publications obtained in the literature search and independently selected the relevant publications. Full-text versions of the remaining publications were assessed for eligibility according to the prespecified eligibility criteria. Discrepancies were addressed through discussion between the two reviewers or, if necessary, by consensus with a third reviewer (SL).

### Data Extraction

The following information was collected from included studies: 1) study characteristics and publication details, including first author name, year of publication, study design, region, sample size in each arm, and median follow-up duration; 2) baseline characteristics, including age, sex, body mass index (BMI), HbA1c level, and fasting glucose level; 3) preparation, dose, intervention, and control; and 4) outcomes of interest as stated above.

If a published study did not report the outcome information but the corresponding registry report from ClinicalTrials.gov included the relevant data, data from the registry report were used. The unit of the blood glucose level was expressed in mmol/L; data expressed as mg/dL were converted into mmol/L using the equation 1 mmol/L = 18 mg/dL.

### Risk of Bias Assessment

Two reviewers (JT and YW) assessed the risk of bias using the version 2 of the Cochrane risk-of-bias tool for randomized trials (ROB 2) and the Newcastle-Ottawa Scale (NOS) for observational studies, and discrepancies were resolved by discussion with a third reviewer (SL) (Higgins et al., 2011). The ROB 2 tool for RCTs covers five types of bias, including randomization, deviations, missing outcome, measurement of the outcome, and selection of the report bias. The NOS consists of eight items to assess the risk of bias in cohort studies (Guyatt et al., 2008).

### Data Synthesis and Statistical Analysis

We evaluated appropriateness for quantitative synthesis for the homogeneity of outcome reporting in the present analysis. For cohort studies, crude and fully adjusted effects were pooled in the quantitative synthesis.

All dichotomous outcomes were measured using relative risks (RRs) in RCTs. We used mean differences (MDs) for continuous outcomes of change from baseline. Gastrointestinal events at the study level were described using both event numbers and proportions, and were pooled when homogeneous. All pooled outcomes were measured using point estimates and corresponding 95% confidence intervals (CIs).

A random effects model using the Mantel-Haenszel method was used to pool study-level effects for RCTs. When the studies involved zero event in one of two groups, the 0.5 correction was used for both groups. We set zero weight for the studies with zero event in both groups in the analysis. Statistical heterogeneity was tested using a χ^2^ test and was quantified as I^2^ statistics. Heterogeneity was considered for a *p* value <0.1 or I^2^ > 50%.

Subgroup analyses were performed according to the following hypotheses: 1) for duration of follow-up (shorter than 24 vs. 24 weeks or longer): larger effects were hypothesized in studies with longer duration, and 2) risk of bias (high vs. low): larger effects were hypothesized in trials with high risk of bias (with one or more high-risk item).

We used the Grading of Recommendation, Assessment, Development, and Evaluation (GRADE) to assess the quality of evidence (Sun et al., 2012). Publication bias was assessed using funnel plots, Begg’s rank correlations, and Egger’s linear regression in cases where at least 10 studies were available. All data analyses were performed using RStudio (R Pack 3.6.1).

## Results

**FIGURE 1:**
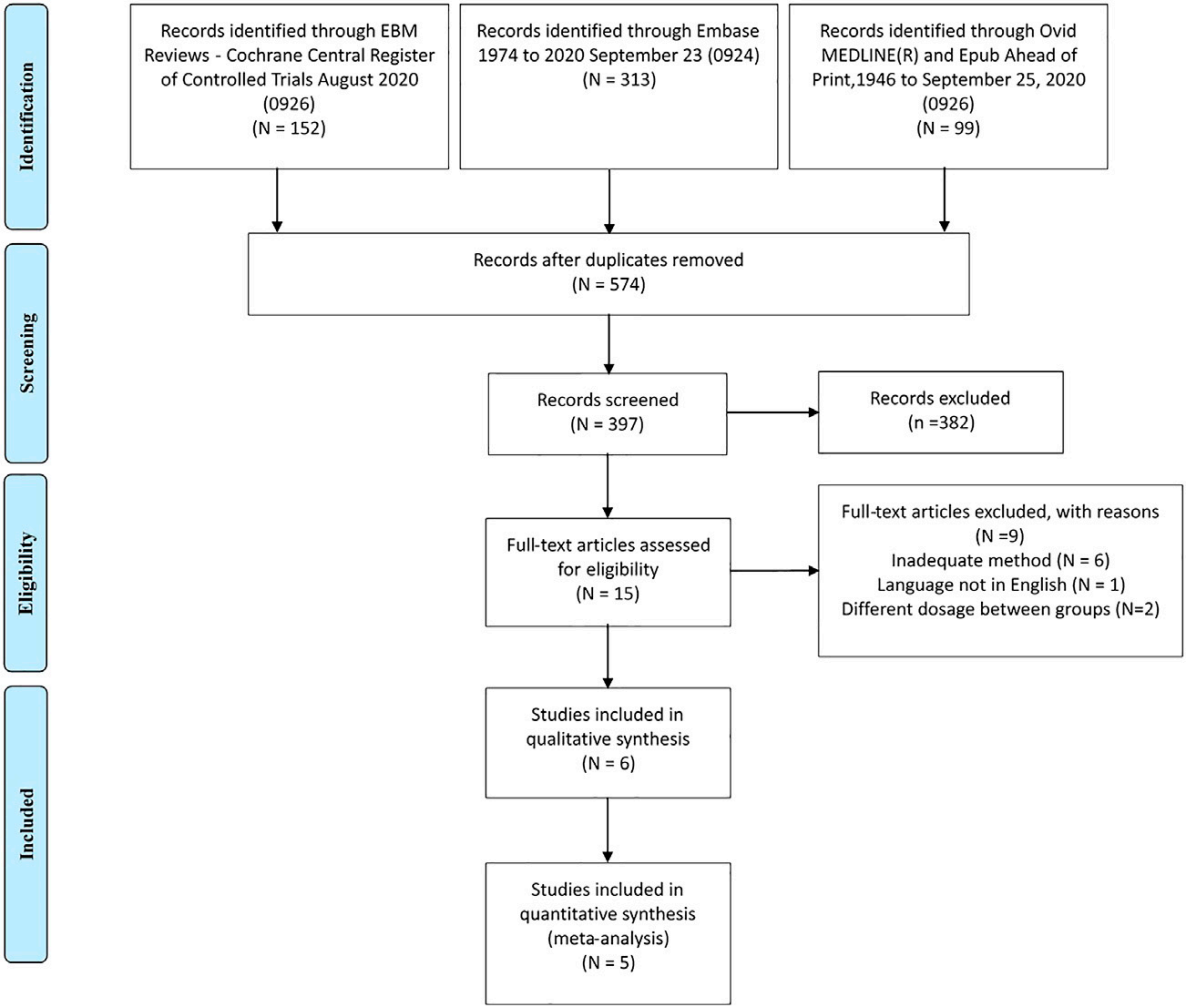
Flow diagram.

As shown in Figure 1, we included five RCTs comprising 1,662 patients (Schwartz et al., 2006; Gao et al., 2008; Hameed et al., 2017; Aggarwal et al., 2018; Ji et al., 2018) and one observational study from the 574 studies identified during the literature search (Donnelly et al., 2009). The characteristics of included randomized control trials are shown in Table 1, and the baseline data of patients in these studies are shown in Supplementary Table S1. One of the included trials was a global multicenter study, and two of the trials were conducted in China. The observational study was conducted retrospectively using electronic medical records in Scotland. The follow-up duration in the included RCTs ranged from 12 to 24 weeks, and the dose of metformin administered to patients ranged from 1,500 to 2000 mg/d. In the included RCTs, the average patient age was 54.39 years, the average BMI was 30.04 kg/m^2^, and 54.69% of patients were male. The mean baseline HbA1c and FPG values were 7.89 and 8.96 mmol/L, respectively.

**TABLE 1:** Characteristics of included randomized control trials.

Trial	Location	Centers	Funding	Randomized (I/C) (n)	Follow-up	Duration of study treatment	Treatment	Control
Aggarwal 2017	North America, Europe, South Africa	In 148 different sites (multicenter)	AstraZeneca	283/285^a^	24 w	24 w	Metformin XR 2000 mg QD	Metformin IR 1000 mg bid
Gao 2008	China	3 lefts in Beijing (multicenter)	National 973 Program of China (2006CB503908), the Natural Science Foundation of Beijing City (7062067) and the National Natural Science Foundation of China (30771032, 30700879)	75/75	12 W	12 W	1,500 mg metformin XR once daily after the dinner	Metformin IR 500 mg thrice daily after meals
Hameed 2017	Pakistan	Medical and endocrinology OPDs of Jinnah Hospital Lahore (single center)	NR	30/30	12 W	12 W	Metformin XR 1000 mg twice daily	Metformin IR 1000 mg twice daily
Ji 2017	China	Multicenter	Merck Serono China Co. Ltd	265/267	18 W	16 W	Metformin extended-release (XR) tablets, orally QD (starting dose 500 mg, maximum treatment dose 2000 mg)	Metformin immediate release (IR) tablets, orally once daily (QD) (starting dose 500 mg, maximum treatment dose 2000 mg)
Schwartz 2006	United States	85 centers in United States (multicenter)	Depomed	178/174	24 W	24 W	1,500 mg extended-release metformin QD	1,500 mg immediate-release metformin twice daily (500 mg in the morning and 1,000 mg in the evening)

a 29 randomized patients were excluded for study site noncompliance; thus, efficacy endpoints were analyzed in a smaller dataset (268/271).

QD, once a day; bid, twice a day; W, week; XR, extended release; IR, immediate release.

The observational study included 137 patients who were receiving metformin XR at the beginning of the study and 10,772 who had been prescribed metformin IR at the beginning of the study. The mean age of the cohort was 62.7 years, and 53% of patients were male.

### Risk of Bias

The risk of bias in the included RCTs is shown in Supplementary Figures S1, S2. Three trials had an open-label design and were evaluated as having a high risk of bias on measurement of the outcome because of insufficient blinding of participants, and the outcome assessments were based on personal judgment (Gao et al., 2008; Hameed et al., 2017; Ji et al., 2018). Furthermore, one trial was rated high risk on deviation bias in the effect of assignment to intervention (Gao et al., 2008).

The observational study had a score of six on the NOS. It lost two stars due to unclear follow-up duration and inadequate adjustment of potential confounders (Donnelly et al., 2009).

### Meta-Analysis of Randomized Control Trials

**FIGURE 2:**
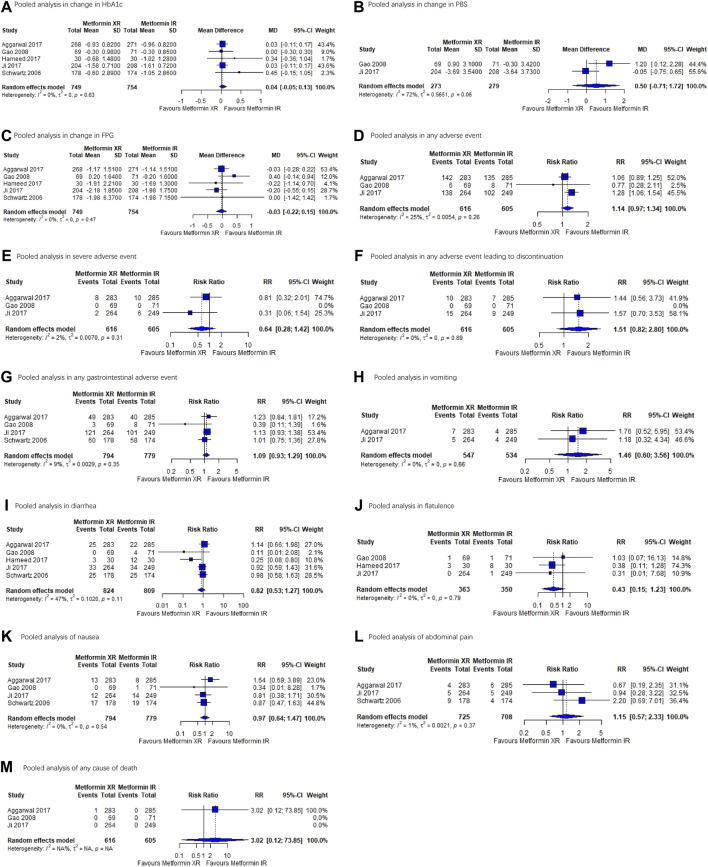
Forest plots for each outcome. **(A)** Pooled analysis in change in HbA1c. **(B)** Pooled analysis in PBS. **(C)** Pooled analysis in FPG. **(D)** Pooled analysis in any adverse event. **(E)** Pooled analysis in severe adverse event. **(F)** Pooled analysis in adverse effect leading to discontinuation. **(G)** Pooled analysis in any gastrointestinal adverse event. **(H)** Pooled analysis in vomiting. **(I)** Pooled analysis in diarrhea. **(J)** Pooled analysis of flatulence. **(K)** Pooled analysis of nausea. **(L)** Pooled analysis of abdominal pain. **(M)** Pooled analysis of any cause of death. Abbreviations: HbA1c, hemoglobin A1C; PBS, postprandial blood glucose; FPG, fasting plasma glucose.

The findings of the quantitative analyses are summarized in Table 2. There was very low– to moderate-level evidence for a lack of difference between metformin XR and IR in adverse events of abdominal pain (RR, 1.15, 95% CI 0.57–2.33), all-cause death (RR, 3.02, 95% CI 0.12–73.85), any adverse events (RR, 1.14, 95% CI 0.97-1.34), any adverse events leading to discontinuation (RR, 1.51, 95% CI 0.82–2.8), any gastrointestinal adverse events (RR, 1.09, 95% CI 0.93–1.29), diarrhea (RR, 0.82, 95% CI 0.53–1.27), flatulence (RR, 0.43, 95% CI 0.15–1.23), nausea (RR, 0.97, 95% CI 0.64–1.47), severe adverse events (RR, 0.64, 95% CI 0.28–1.42), and vomiting (RR, 1.46, 95% CI 0.6–3.56). In the pooled analysis, no statistical difference was observed in change in HbA1c (MD 0.04%, 95% CI −0.05–0.13%), postprandial blood glucose (MD 0.50 mmol/L, 95% CI −0.71–1.72 mmol/L), and FPG (−0.03 mmol/L, 95% CI −0.22–0.15 mmol/L). Forest plots of all endpoints are shown in Figures 2A–M. Our study did not identify any subgroup effects among the outcomes (Supplementary Tables S2, S3).

**TABLE 2:** Summary of findings.

Outcomes	Number of studies	Number of participants	Design	Risk of bias	Inconsistency	Indirectness	Imprecision	Others	Event rate in the metformin XR arm	Event rate in the metformin IR arm	Relative risks/mean difference	Quality of evidence
Abdominal pain	3	1,433	RCTs	Serious risk of bias	No serious inconsistency	No serious indirectness	No serious imprecision	None	18/725 (2.5%)	15/708 (2.1%)	1.15 (0.57–2.33)	Moderate
All-cause death	3	1,221	RCTs	Serious risk of bias	No serious inconsistency	No serious indirectness	Very serious imprecision^a^	None	1/616 (0.2%)	0/605 (0%)	3.02 (0.12–73.85)	Very low
Any adverse events	3	1,221	RCTs	Serious risk of bias	No serious inconsistency	Serious indirectness^b^	Serious imprecision	None	286/616 (46.4%)	245/605 (40.5%)	1.14 (0.97–1.34)	Very low
Any adverse events leading to discontinuation	3	1,221	RCTs	Serious risk of bias	No serious inconsistency	Serious indirectness^b^	No serious imprecision	None	25/616 (4.1%)	16/605 (2.6%)	1.51 (0.82–2.8)	Low
Any gastrointestinal adverse events	4	1,573	RCTs	Serious risk of bias	No serious inconsistency	No serious indirectness	No serious imprecision	None	233/794 (29.3%)	207/779 (26.6%)	1.09 (0.93–1.29)	Moderate
Diarrhea	5	1,633	RCTs	Serious risk of bias	No serious inconsistency	No serious indirectness	No serious imprecision	None	86/824 (10.4%)	97/809 (12%)	0.82 (0.53–1.27)	Moderate
Flatulence	3	713	RCTs	Serious risk of bias	No serious inconsistency	No serious indirectness	No serious imprecision	None	4/363 (1.1%)	10/350 (2.9%)	0.43 (0.15–1.23)	Moderate
Nausea	4	1,573	RCTs	Serious risk of bias	No serious inconsistency	No serious indirectness	No serious imprecision	None	42/794 (5.3%)	42/779 (5.4%)	0.97 (0.64–1.47)	Moderate
Severe adverse events	3	1,221	RCTs	Serious risk of bias	No serious inconsistency	Serious indirectness^b^	No serious imprecision	None	10/616 (1.6%)	16/605 (2.6%)	0.64 (0.28–1.42)	Low
Vomiting	2	1,081	RCTs	Serious risk of bias	No serious inconsistency	No serious indirectness	No serious imprecision	None	12/547 (2.2%)	8/534 (1.5%)	1.46 (0.6–3.56)	Moderate
Change in HbA1c	5	1,503	RCTs	No serious risk of bias	No serious inconsistency	Serious indirectness^b^	No serious imprecision	None	—	—	0.04 (−0.05–0.13)	Moderate
Change in PBS (mmol/L)	2	552	RCTs	No serious risk of bias	No serious inconsistency	Serious indirectness^b^	No serious imprecision	None	—	—	0.50 (−0.71–1.72)	Moderate
Change in FPG (mmol/L)	5	1,503	RCTs	No serious risk of bias	No serious inconsistency	Serious indirectness^b^	No serious imprecision	None	—	—	−0.03 (−0.22–0.15)	Moderate

RCT, randomized control trial; NA, not available; HbA1c, hemoglobin A1C; PBS, postprandial blood sugar; FPG, fasting plasma glucose; XR, extended release; IR, immediate release.

a Imprecision of evidence was downgraded due to wide 95% CI which could not support clinical decision-making.

b Indirectness of evidence was downgraded due to composite outcomes or surrogate outcomes which is indirect to the patients.

Indirectness of evidence was downgraded for any adverse events, any adverse events leading to discontinuation, severe adverse events, change in HbA1c, postprandial blood glucose, and FPG due to the indirectness of composite outcomes or surrogate outcomes. Furthermore, imprecision of evidence was downgraded for all-cause death and any adverse events because of wide 95% CIs that could not be used to support clinical decision-making.

### Compliance and Satisfaction

Three of the included RCTs investigated patient compliance to treatment. Quantitative analysis of these assessments was not performed because of heterogeneity in the measurements. In an RCT conducted in 268 patients in 2017, one patient in the metformin XR group discontinued treatment because of noncompliance, whereas no patients discontinued in the metformin IR group (Aggarwal et al., 2018). An RCT conducted in 2008 reported that 97.2% of tablets were taken in the metformin XR group and 93.8% of tablets were taken in the metformin IR group (Gao et al., 2008); however, the investigators did not perform a statistical analysis in that study. Another RCT conducted in 2018 reported that 255 of 264 (96.6%) and 247 of 261 (94.6%) patients achieved good treatment compliance in the metformin XR and metformin IR groups, respectively (Ji et al., 2018). In the included observational study, compliance to metformin XR (80%) was significantly higher than that observed for metformin IR (72%). Patient compliance markedly increased after changing from metformin IR (62%) to metformin XR (81%) (Donnelly et al., 2009). None of the included studies compared patient satisfaction with metformin XR vs. metformin IR.

## Discussion

Our systematic review did not identify clear differences in the effectiveness and safety of metformin XR vs. metformin IR with very low to moderate certainty, although metformin XR use was more likely to be associated with treatment compliance. To the best of our knowledge, this is the first systematic review to compare the IR and XR preparations of metformin.

Although both metformin IR and XR are widely used in clinical practice, the rationale for choosing one formulation over the other has not been widely examined. Guidance on the differential use of metformin XR vs. IR is largely absent in clinical practice guidelines for diabetes treatment, with the exception of the NICE guideline, which recommends the use of metformin XR in patients intolerant to metformin IR (McGuire et al., 2016). However, the NICE guideline recommendation is not supported by any specific evidence. Although the findings of some previous studies are in alignment with the NICE recommendation, the results from those studies are not conclusive (Derosa et al., 2017; Henry et al., 2018). In one such RCT, a reduced risk of gastrointestinal intolerance was observed with metformin XR treatment. In that study, the metformin XR dose was systematically lower than that of metformin IR, and serum metformin levels in patients receiving metformin XR were almost 50% less than those in patients receiving metformin IR (Henry et al., 2018). Although the glucose-lowering effect of metformin XR was inferior to that of metformin IR, the results of this trial, in which the ratio of the glucose level decrease to the mean serum level of metformin was calculated, indicate the superior efficacy of metformin XR (Henry et al., 2018). A study conducted in Italy also supported the superior glucose-lowering effect of metformin XR at the maximal tolerated dose compared with metformin IR (Derosa et al., 2017). Of note, metformin XR was associated with significantly fewer adverse events than metformin IR at a 50% lower average maintenance dose (XR 1000 ± 500 mg vs. IR 2000 ± 1,000 mg), meaning that most patients receiving metformin XR stopped up-titration before reaching the maximal dose (Derosa et al., 2017). However, the data from these trials did not clarify whether the superior tolerance observed for metformin XR was attributable to the lower dose. Neither of these trials was included in our systematic review because both featured different doses of metformin XR and IR between the treatment arms.

Our findings support similar effectiveness and safety of metformin XR and IR, indicating that it might not be appropriate to switch from metformin IR to metformin XR for the purpose of improving glucose control or reducing adverse events. Our results highlight the need for a change in clinical practice with respect to the selection of metformin preparation.

Our findings also indicate better compliance with metformin XR treatment due to its once-daily dosing regimen. The results were consistent across the included randomized trials and observational studies, regardless of the population heterogeneity compared with real-world practice (Zhou et al., 2021). We inferred that as the number of doses increases, patients are increasingly likely to miss a dose, leading to noncompliance (Godman et al., 2020). This observation is not unique to metformin therapy in diabetes but is also observed in the treatment of other chronic diseases (Shahiwala, 2011). A 2015 meta-analysis indicated that once-daily administration of antibiotics was associated with better compliance than twice- or thrice-daily treatment (Falagas et al., 2015). Another meta-analysis of antiviral treatment of HIV reported that compliance to a once-daily regimen was slightly better than that to a twice-daily regimen (Nachega et al., 2014). Further, a pilot study among 110 patients reported that once-daily dosage regimens were largely preferred by patients (Witticke et al., 2012). Therefore, clinicians should consider patient preference when deciding on the type of formulation to prescribe and should consider metformin XR for patients who prefer once-daily dosing. The selection of metformin preparation thus represents a good example of patient-centered care, where shared decision-making can be beneficial. Clinicians should also consider which patients will benefit most from once-daily administration. For example, adults in full-time employment may benefit from less frequent dosing, while it may not be necessary to prescribe metformin XR to patients with polypharmacy.

Our previous survey study indicated that most outpatients in China did not have an accurate understanding of why they were receiving the XR formulation of metformin (Liu et al., 2021). The majority (81.2%) believed that metformin XR was more effective and tolerable than metformin IR (Liu et al., 2021). This finding when considered alongside the results of the current systematic review is indicative of shortcomings in the dissemination of evidence to the patients and, potentially, to clinicians. The findings of our systematic review support the education of patients regarding the use of antidiabetic medications.

Our study had some limitations. First, we included only five randomized trials and one observational study; however, the overall sample size of more than 10,000 patients supported the robustness of our results. Second, long-term endpoints were not identified for metformin XR and IR use. Further long-term observational studies are needed to confirm the present findings.

In conclusion, although metformin XR and IR formulations have similar effectiveness and safety, metformin XR is associated with increased treatment compliance. These findings require dissemination to patients and clinicians, and long-term observation of the use of these two formulations is warranted.

## Data Availability

The original contributions presented in the study are included in the article/Supplementary Material; further inquiries can be directed to the corresponding authors.
