# Supplementary material for: Long-Acting Metformin Vs. Metformin Immediate Release in Patients With Type 2 Diabetes: A Systematic Review
Source: Front Pharmacol. 2021 May 17;12:669814. doi: 10.3389/fphar.2021.669814 (PMC8165304; doi:10.3389/fphar.2021.669814)
Supplement: Supplementary file 1 [file DataSheet1.docx]

**Table S1 Baseline characteristics of patients in the included studies**

| Trial | Age, year | Male, n (%) | BMI, kg/m^2^ | HbA1c (Metformin XR), % | HbA1c (metformin IR), % | FPG (Metformin XR), mmol/L | FPG (Metformin IR), mmol/L | PBS (Metformin XR), mmol/L | PBS (Metformin IR), mmol/L |
| --- | --- | --- | --- | --- | --- | --- | --- | --- | --- |
| Aggarwal 2017 | 56.05 | 295(54.8%) | 32.85 | 7.58± 0.6 | 7.76±0.5 | 8.54±1.71§ | 8.77±1.83§ | NA | NA |
| Gao 2008 | 54.60 | 75(50.0%) | 26.30 | 6.5±1.1 | 6.5±1.0 | 6.91±1.30 | 7.0±1.8 | 10.2±3.1 | 10.4±3.3 |
| Hameed 2017 | 45.99 | 49(54%) | 25.98 | 7.32±1.533 | 7.42±1.299 | 9.86±2.47§ | 9.22±1.32§ | NA | NA |
| Ji 2017 | 53.75 | 312(58.6%) | 26.20 | 8.2±0.9 | 8.2±0.8 | 8.7±2.10 | 8.5±1.7 | 14.8±3.8 | 14.6±3.4 |
| Schwartz 2006 | 54.00 | 178(50.6%) | 33.60 | 8.22±3.34# | 8.70±3.30# | 10.56±7.34# § | 10.92±8.21#§ | NA | NA |
| Donnelly 2009  (Observational study) | 62.70 | 5771(52.90%) | 31.31 | 8.7 ±1.7 | 8.9 ±1.7 | NA | NA | NA | NA |

Abbreviations: BMI, body mass index; HbA1c, hemoglobin A1c; FPG, fasting plasma glucose; PBS, postprandial blood sugar (glucose); Metformin XR, extended-release Metformin; Metformin IR, immediate-release Metformin.

#SDs were converted from SEs.

§Formula to calculate mmol/l from mg/dl for blood glucose: mmol/l=mg/dl/18.

**Table S2 Summary of the subgroup analyses of safety outcomes**

| Outcomes | Subgroups | Categories | RR | P value | Interactive Q value | Interactive P value |
| --- | --- | --- | --- | --- | --- | --- |
| Abdominal pain | Follow-up duration | ≥24 weeks | 1.25 (0.39~3.98) | 0.71 | 0.10 | 0.75 |
|  |  | <24 weeks | 0.94 (0.28~3.22) | 0.93 |  |  |
|  | Risk of bias | Low | 1.25 (0.39~3.98) | 0.71 | 0.10 | 0.75 |
|  |  | High | 0.94 (0.28~3.22) | 0.93 |  |  |
| All-cause death | Follow-up duration | ≥24 weeks | 3.02 (0.12~73.85) | 0.50 | 0.00 | NA |
|  |  | <24 weeks | NA | NA |  |  |
|  | Risk of bias | Low | 3.02 (0.12~73.85) | 0.50 | 0.00 | NA |
|  |  | High | NA | NA |  |  |
| Any adverse events | Follow-up duration | ≥24 weeks | 1.06 (0.89~1.25) | 0.50 | 1.75 | 0.19 |
|  |  | <24 weeks | 1.25 (1.04~1.51) | 0.02 |  |  |
|  | Risk of bias | Low | 1.06 (0.89~1.25) | 0.50 | 1.75 | 0.19 |
|  |  | High | 1.25 (1.04~1.51) | 0.02 |  |  |
| Any adverse events leading to discontinuation | Follow-up duration | ≥24 weeks | 1.44 (0.56~3.73) | 0.45 | 0.02 | 0.89 |
|  |  | <24 weeks | 1.57 (0.70~3.53) | 0.27 |  |  |
|  | Risk of bias | Low | 1.44 (0.56~3.73) | 0.45 | 0.02 | 0.89 |
|  |  | High | 1.57 (0.70~3.53) | 0.27 |  |  |
| Any gastrointestinal adverse events | Follow-up duration | ≥24 weeks | 1.09 (0.86~1.37) | 0.48 | 0.35 | 0.55 |
|  |  | <24 weeks | 0.80 (0.30~2.16) | 0.66 |  |  |
|  | Risk of bias | Low | 1.09 (0.86~1.37) | 0.48 | 0.35 | 0.55 |
|  |  | High | 0.8 (0.30~2.16) | 0.66 |  |  |
| Diarrhea | Follow-up duration | ≥24 weeks | 1.05 (0.72~1.53) | 0.79 | 1.88 | 0.17 |
|  |  | <24 weeks | 0.44 (0.13~1.45) | 0.18 |  |  |
|  | Risk of bias | Low | 0.79 (0.41~1.52) | 0.48 | 0.16 | 0.69 |
|  |  | High | 0.53 (0.09~3.28) | 0.50 |  |  |
| Flatulence | Follow-up duration | <24 weeks | 0.43 (0.15~1.23) | 0.11 | 0.00 | NA |
|  | Risk of bias | High | 0.62 (0.08~5.00) | 0.65 | 0.17 | 0.68 |
|  |  | Low | 0.38 (0.11~1.28) | 0.12 |  |  |
| Nausea | Follow-up duration | ≥24 weeks | 1.11 (0.61~2.01) | 0.73 | 0.56 | 0.45 |
|  |  | <24 weeks | 0.77 (0.37~1.61) | 0.49 |  |  |
|  | Risk of bias | Low | 1.11 (0.61~2.01) | 0.73 | 0.56 | 0.45 |
|  |  | High | 0.77 (0.37~1.61) | 0.49 |  |  |
| Severe adverse events | Follow-up duration | ≥24 weeks | 0.81 (0.32~2.01) | 0.64 | 1.01 | 0.31 |
|  |  | <24 weeks | 0.31 (0.06~1.54) | 0.15 |  |  |
|  | Risk of bias | Low | 0.81 (0.32~2.01) | 0.64 | 1.01 | 0.31 |
|  |  | High | 0.31 (0.06~1.54) | 0.15 |  |  |
| Vomiting | Follow-up duration | ≥24 weeks | 1.76 (0.52~5.95) | 0.36 | 0.20 | 0.66 |
|  |  | <24 weeks | 1.18 (0.32~4.34) | 0.80 |  |  |
|  | Risk of bias | Low | 1.76 (0.52~5.95) | 0.36 | 0.20 | 0.66 |
|  |  | High | 1.18 (0.32~4.34) | 0.80 |  |  |

Abbreviations: RR, relative risk; NA, not available.

**Table S3 Summary of the subgroup analyses of efficacy outcomes**

| Outcome | Subgroups | Categories | MD | P value | Interactive Q value | Interactive P value |
| --- | --- | --- | --- | --- | --- | --- |
| Change in FPG | Risk of bias | Low | -0.04 (-0.28~0.20) | 0.73 | 0.11 | 0.74 |
|  |  | High | 0.06 (-0.52~0.65) | 0.83 |  |  |
|  | Follow-up duration | ≥24 weeks | -0.03 (-0.28~0.22) | 0.82 | 0.01 | 0.91 |
|  |  | <24 weeks | -0.00 (-0.42~0.42) | 1.00 |  |  |
| Change in HbA1c | Risk of bias | Low | 0.11 (-0.11~0.34) | 0.33 | 0.44 | 0.51 |
|  |  | High | 0.02 (-0.1~0.15) | 0.70 |  |  |
|  | Follow-up duration | ≥24 weeks | 0.13 (-0.22~0.49) | 0.46 | 0.27 | 0.60 |
|  |  | <24 weeks | 0.03 (-0.09~0.16) | 0.58 |  |  |
| Change in PBS | Risk of bias | High | 0.50 (-0.71~1.72) | 0.42 | 0 | NA |
|  | Follow-up duration | <24 weeks | 0.50 (-0.71~1.72) | 0.42 | 0 | NA |

Abbreviations: HbA1c, hemoglobin A1c; FPG, fasting plasma glucose; PBS, postprandial blood sugar (glucose); MD, mean difference; NA, not available.

**Figure S1-1 Risk of Bias graph of safety outcomes**


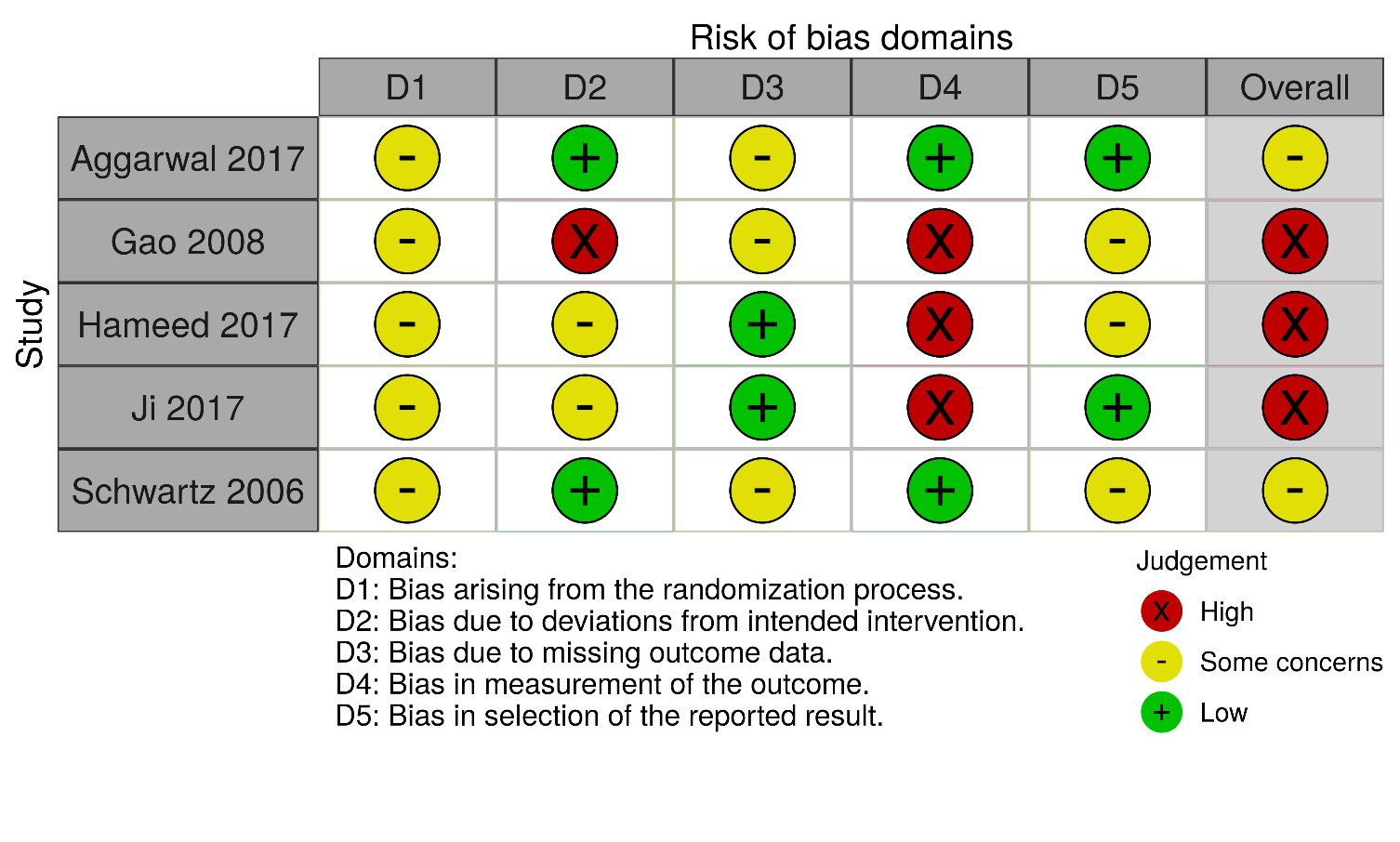


**Figure S1-2 Risk of Bias graph of efficacy outcomes**


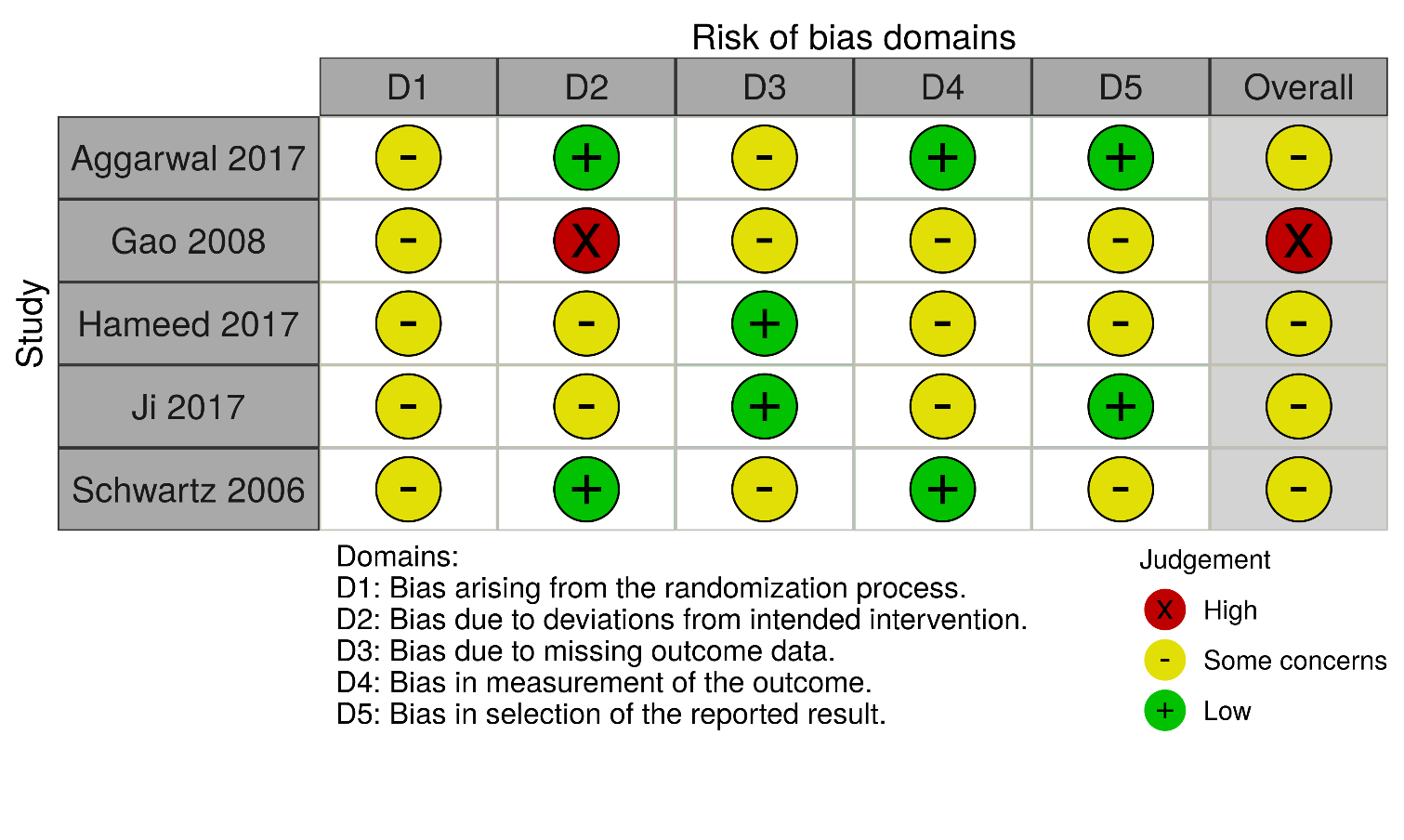


**Figure S2-1 Risk of bias summary of safety outcomes**


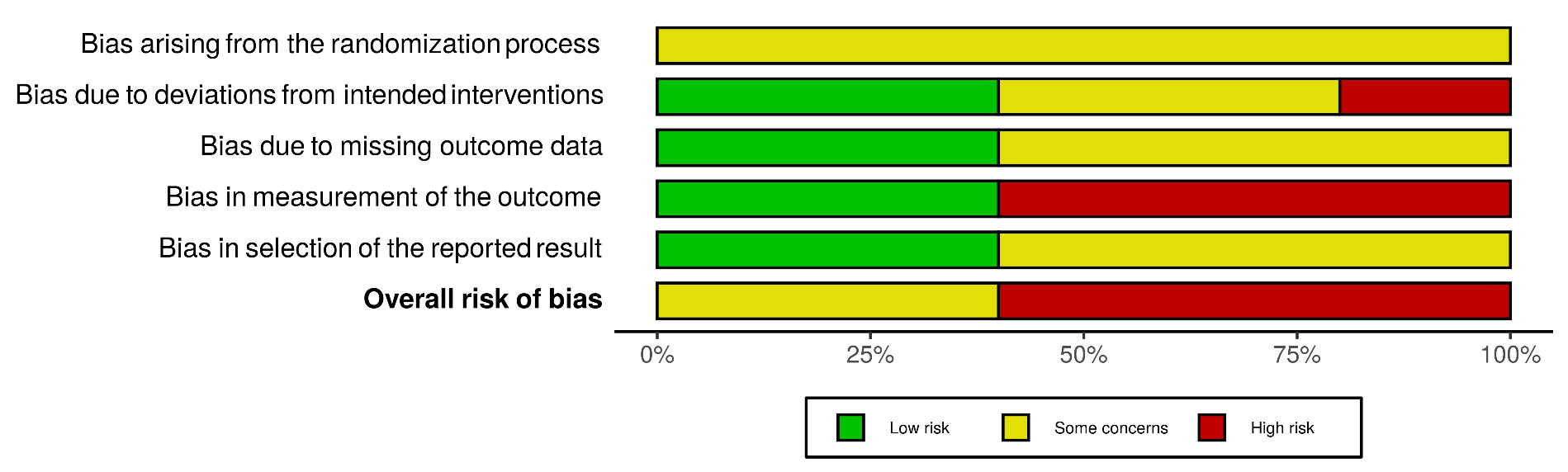


**Figure S2-2 Risk of bias summary of efficacy outcomes**


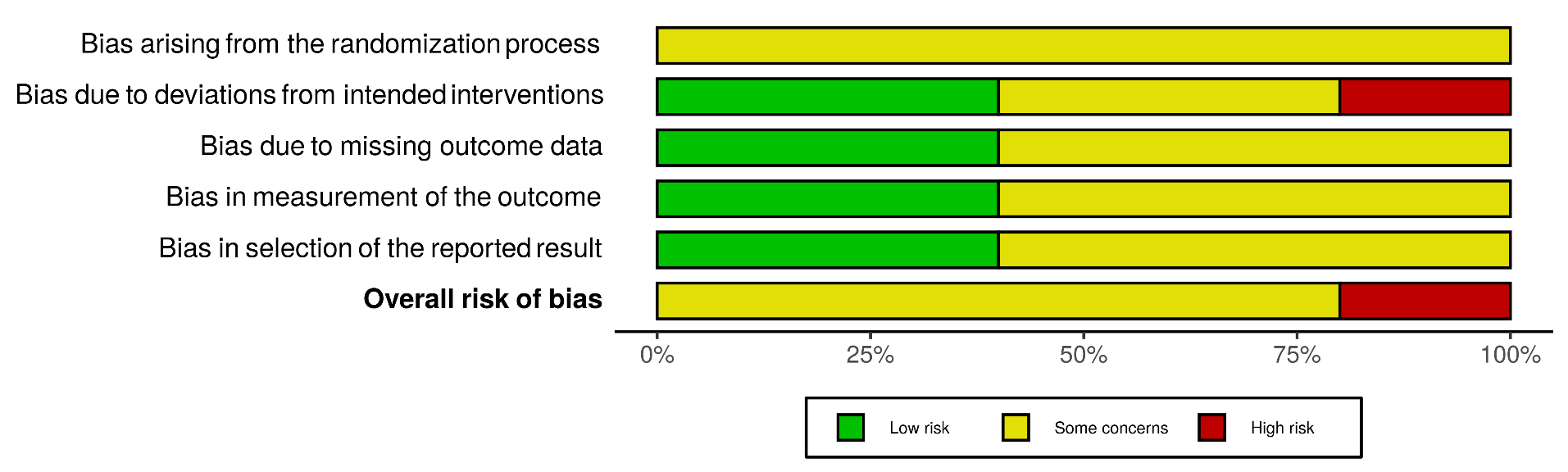


**Supplementary Material: Searching strategy**

EBM Reviews - Cochrane Central Register of Controlled Trials August 2020

1. diabetes.mp. or exp diabetes mellitus/

2. diabetic.mp.

3. DM.mp.

4. 1 or 2 or 3

5. glucophage XR.mp.

6. glumetza.mp.

7. fortamet.mp.

8. glucophage SR.mp.

9. glucophage MR.mp.

10. Riomet ER.mp.

11. sukkarto SR.mp.

12. diagemet XL.mp.

13. metformin ER.mp.

14. metformin XR.mp.

15. metformin extended release.mp.

16. extended-release metformin.mp.

17. metformin prolonged release.mp.

18. prolonged-release metformin.mp.

19. metformin sustained release.mp.

20. sustained-release metformin.mp.

21. metformin sustained action.mp.

22. sustained-action metformin.mp.

23. metformin controlled release.mp.

24. controlled-release metformin.mp.

25. metformin controlled delivery.mp.

26. controlled-delivery metformin.mp.

27. depot metformin.mp.

28. metformin modified release.mp.

29. modified-release metformin.mp.

30. long-acting metformin.mp.

31. metformin timed release.mp.

32. timed-release metformin.mp.

33. metformin delayed release.mp.

34. delayed-release metformin.mp.

35. 5 or 6 or 7 or 8 or 9 or 10 or 11 or 12 or 13 or 14 or 15 or 16 or 17 or 18 or 19 or 20 or 21 or 22 or 23 or 24 or 25 or 26 or 27 or 28 or 29 or 30 or 31 or 32 or 33 or 34

36. 4 and 35

Embase 1974 to 2020 September 23

1. diabetes.mp. or exp diabetes mellitus/

2. diabetic.mp.

3. DM.mp.

4. 1 or 2 or 3

5. glucophage XR.mp.

6. glumetza.mp.

7. fortamet.mp.

8. glucophage SR.mp.

9. glucophage MR.mp.

10. Riomet ER.mp.

11. sukkarto SR.mp.

12. diagemet XL.mp.

13. metformin ER.mp.

14. metformin XR.mp.

15. metformin extended release.mp.

16. extended-release metformin.mp.

17. metformin prolonged release.mp.

18. prolonged-release metformin.mp.

19. metformin sustained release.mp.

20. sustained-release metformin.mp.

21. metformin sustained action.mp.

22. sustained-action metformin.mp.

23. metformin controlled release.mp.

24. controlled-release metformin.mp.

25. metformin controlled delivery.mp.

26. controlled-delivery metformin.mp.

27. depot metformin.mp.

28. metformin modified release.mp.

29. modified-release metformin.mp.

30. long-acting metformin.mp.

31. metformin timed release.mp.

32. timed-release metformin.mp.

33. metformin delayed release.mp.

34. delayed-release metformin.mp.

35. 5 or 6 or 7 or 8 or 9 or 10 or 11 or 12 or 13 or 14 or 15 or 16 or 17 or 18 or 19 or 20 or 21 or 22 or 23 or 24 or 25 or 26 or 27 or 28 or 29 or 30 or 31 or 32 or 33 or 34

36. 4 and 35

Ovid MEDLINE(R) and Epub Ahead of Print, In-Process & Other Non-Indexed Citations, Daily and Versions(R) 1946 to September 25, 2020

1. diabetes.mp. or exp diabetes mellitus/

2. diabetic.mp.

3. DM.mp.

4. 1 or 2 or 3

5. glucophage XR.mp.

6. glumetza.mp.

7. fortamet.mp.

8. glucophage SR.mp.

9. glucophage MR.mp.

10. Riomet ER.mp.

11. sukkarto SR.mp.

12. diagemet XL.mp.

13. metformin ER.mp.

14. metformin XR.mp.

15. metformin extended release.mp.

16. extended-release metformin.mp.

17. metformin prolonged release.mp.

18. prolonged-release metformin.mp.

19. metformin sustained release.mp.

20. sustained-release metformin.mp.

21. metformin sustained action.mp.

22. sustained-action metformin.mp.

23. metformin controlled release.mp.

24. controlled-release metformin.mp.

25. metformin controlled delivery.mp.

26. controlled-delivery metformin.mp.

27. depot metformin.mp.

28. metformin modified release.mp.

29. modified-release metformin.mp.

30. long-acting metformin.mp.

31. metformin timed release.mp.

32. timed-release metformin.mp.

33. metformin delayed release.mp.

34. delayed-release metformin.mp.

35. 5 or 6 or 7 or 8 or 9 or 10 or 11 or 12 or 13 or 14 or 15 or 16 or 17 or 18 or 19 or 20 or 21 or 22 or 23 or 24 or 25 or 26 or 27 or 28 or 29 or 30 or 31 or 32 or 33 or 34

36. 4 and 35
